# Plasma fibrin D-dimer and the risk of left atrial thrombus: A systematic review and meta-analysis

**DOI:** 10.1371/journal.pone.0172272

**Published:** 2017-02-16

**Authors:** Huaibin Wan, Shuang Wu, Yanmin Yang, Jun Zhu, Aidong Zhang, Yan Liang

**Affiliations:** 1 Department of Cardiology, the First Affiliated Hospital, Jinan University, Guangzhou, China; 2 Emergency and Intensive Care Center, Fuwai Hospital, National Center for Cardiovascular Diseases, Chinese Academy of Medical Sciences & Peking Union Medical College, Beijing, China; Maastricht University Medical Center, NETHERLANDS

## Abstract

**Background:**

Plasma fibrin d-dimer has been taken as a marker for thrombus. The aim of this study was to evaluate the relationship between d-dimer (DD) levels and left atrial spontaneous echo contrast (SEC)/left atrial thrombus (LAT).

**Methods:**

We identified clinical studies by systematic search of MEDLINE and EMBASE databases up to Feb 2016. All observational studies that considered DD as a study factor and trans-esophageal echocardiography (TEE) identified SEC/LAT as an outcome were included. Two reviewers independently selected the studies and extracted the data.

**Results:**

Of the 21 included studies, 16 studies (2652 patients) have compared the mean DD differences between patients with and without an evidence of the presence of SEC/LAT, 9 studies (1667 patients) have estimated the diagnostic value of DD in the presence of LAT, and 11 studies (1856 patients) have available information to calculate a ratio of the presence of LAT among individuals in the top and the bottom third of DD levels. The pooled standardized mean difference (SMD) of DD between patients with and without left atrial SEC and/or LAT was 1.29 [95%CI: 0.51, 2.08], with SMDs of 0.42 [95% CI: 0.08, 0.77] and 2.34 [95% CI: 1.01, 3.68] in SEC/LAT and LAT subgroups, respectively. The combined risk ratio of the presence of LAT among individuals between the top of the distribution of DD levels and that in the bottom third was 3.84 [95% CI: 2.35, 6.28], associating with a mean difference of 0.78 ug/ml (1.10 vs 0.32 ug/ml). The pooled sensitivity, specificity and positive likelihood ratio of DD for LAT were 0.75 [95% CI: 0.65, 0.83], 0.81 [95% CI: 0.59, 0.93] and 4.0 [95% CI: 1.7, 9.9], respectively.

**Conclusions:**

High plasma fibrin DD was associated with left atrial SEC/LAT, particularly among patients with LAT. DD levels have moderate sensitivity and specificity for diagnosing LAT.

## Introduction

Elevated plasma fibrin d-dimer (DD) levels suggest the presence of hypercoagulability or prothrombotic state [1]. Thus DD has been taken as a potential non-invasive marker of thrombogenesis and thromboembolism. In clinical settings such as deep venous thrombosis (DVT) [2], pulmonary embolism (PE) [3,4] and acute aortic dissection [5,6], DD has an excellent negative predictive value. Pooled data of 10 studies showed that an elevated DD was independently associated with twice as much increased risk of near-term systemic embolism [7].

Left atrial spontaneous echo contrast (SEC) and left atrial thrombus (LAT), which are identified by trans-esophageal echocardiography (TEE), have been confirmed to be associated with increased thromboembolic risk [8,9,10]. As a semi-invasive procedure, however, TEE can induce obvious discomfort and has potential risk of esophageal damage [11]. Thus, it is hard to apply TEE to all patients with left atrial dysfunction, such as those with atrial fibrillation, atrial flutter and mitral stenosis. Some studies suggested that DD is a valuable parameter to exclude left atrial SEC and LAT [12,13]. However, these studies included a relative small number of patients with inconsistent outcomes. Therefore, we performed a systemic review and meta-analysis to evaluate the association between DD and the presence of LAT/SEC.

## Materials and methods

### Search strategy

We searched the MEDLINE and EMBASE databases for articles published from initiations to Feb. 2016, and identified additional studies by hand-searching references of original articles or review articles on this topic and by personal contact with investigators. The search terms and algorithm for the literature search were as follows: (“fibrin fragment D” [MeSH] OR “d-dimer” OR “d-dimer fibrin” OR “d-dimer fragments” OR “fibrin fragment D1 dimer” OR “fibrin fragment DD” OR “fibin fragment D-dimer”) AND((“atrial function, left”[MeSH] AND (“thromboses” OR “thrombus”[MeSH]) OR “left atrial thrombus” OR “appendage thrombus” OR “spontaneous echo contrast”). The search language was restricted to English.

### Selection of studies

The selection of eligible studies was performed by two independent reviewers (H.W. and S.W.). Inconsistency regarding the selection of studies was resolved by consensus with the senior consultants (Y.Y. and J.Z.). Studies were selected if they met the following criteria: 1) DD was a study factor; 2) the outcome of interest was any type of TEE-identified left atrial SEC or LAT; 3) studies had sufficient data for pooling, i.e., number of subjects, mean and standard deviation (or median and range) of DD between SEC/LAT group and control group for continuous data; or frequencies and cutoff values of DD to predict the presence of LAT for categorical data; 4) studies were not duplicates. Review articles, case-reports and studies with insufficient data for pooling were excluded in case of no response after contacting the authors twice. Left atrial SEC was defined as dynamic smoke-like echoes with characteristic swirling patterns in left atrium or its appendage. LAT was defined as echo-dense intracavitary mass adjacent to the endocardial surface of the left atrium or its appendage and acoustic characteristics that clearly differentiated from such normal structures as endocardium or pectinate muscles.

### Data extraction

Baseline characteristics of included studies (i.e., study settings, period of publishing, country, mean age, percentage of males, history of atrial fibrillation (AF), mitral stenosis, acute ischemic stroke and anticoagulation, instruments for TEE, methods used for measuring DD, excluded population), data of outcomes, i.e., mean DD and standard deviation in two groups, the log ratio of the risk of LAT among individuals in the top third vs those in the bottom third based on the measurement of DD, and the cutoff value of DD selected for maximal sensitivity and the frequency of presenting LAT, were extracted by 2 independent reviewers (H.W. and S.W.) using a standardized data extraction form. Disagreement was resolved by consensus with the senior consultants (Y.Y. and J.Z.). Missing data was obtained by contacting the corresponding authors.

### Risk of bias assessment

Two authors (A.Z. and L.Y.) independently assessed risk of bias of each study. For studies reporting mean DD difference, the quality assessment was conducted via the Newcastle and Ottawa risk of bias criteria [14] for assessing the quality of case-control studies. Three domains were considered, i.e., selection of study groups (4 items), comparability of groups (2 items), and ascertainment of exposure (2 items). Each item was graded either 0 or 1 with a total score ranging from 0 to 8; higher total score reflected higher quality and lower risk of bias. For the quality assessment of studies’ diagnostic accuracy of DD on the prediction of LAT, the bias was assessed via QUADAS-2[15], including four domains in risk of bias, i.e., patient selection, index test, reference standard, and flow and timing, and three domains in applicability, i.e. patient selection, index test and reference standard. Studies were judged as “low”, “high” or “unclear” according to domains relating to risk of bias or applicability. Disagreement was resolved by consensus after discussion with senior consultants (Y.Y. and J.Z).

### Statistical analysis

For continuous outcomes, the mean DD difference between SEC/LAT and control group was estimated for each study and pooled using a standardized mean difference (SMD). If data of DD was reported in the form of median, range and size only, the mean and the variance were converted according to the method reported by Hozo et al.[16] The ratios of risk for the presence of LAT based on the tertiles of DD levels were estimated by the method reported by Danesh J. et al[17]. For dichotomous outcomes, the true positive, true negative, false positive and false negative of a DD cutoff value on the presence of LAT were estimated for each study. Bivariate meta-analysis was applied for pooling diagnostic parameters including sensitivity, specificity, and likelihood ratio positive of DD level on LAT using midas command in STATA.

Heterogeneity of the effect estimates was assessed and quantified using the Q statistic and I^2^ statistic. If significant heterogeneity was detected (P value <0.10 or I^2^≥25%), a random-effect model (Dersimonian & Laird method) was applied; otherwise, a fixed-effect model (inverse-variance method) was used. Sources of heterogeneity were explored by fitting each of the co-variables (i.e. mean age, study settings, sample size, percentage of males, mitral stenosis (Yes/No), AF (percentage), acute ischemic stroke (Yes/No) and anticoagulation (Yes/No), cuffoff value of DD) in meta-regression models. Publication bias was assessed using an Egger’s test and funnel plot. All analyses were performed using STATA software (version 12.0, StataCorp, USA). A two-sided test with P-value <0.05 was considered statistically significant except for the test of heterogeneity, in which a P-value <0.10 was used.

## Results

**Fig 1:**
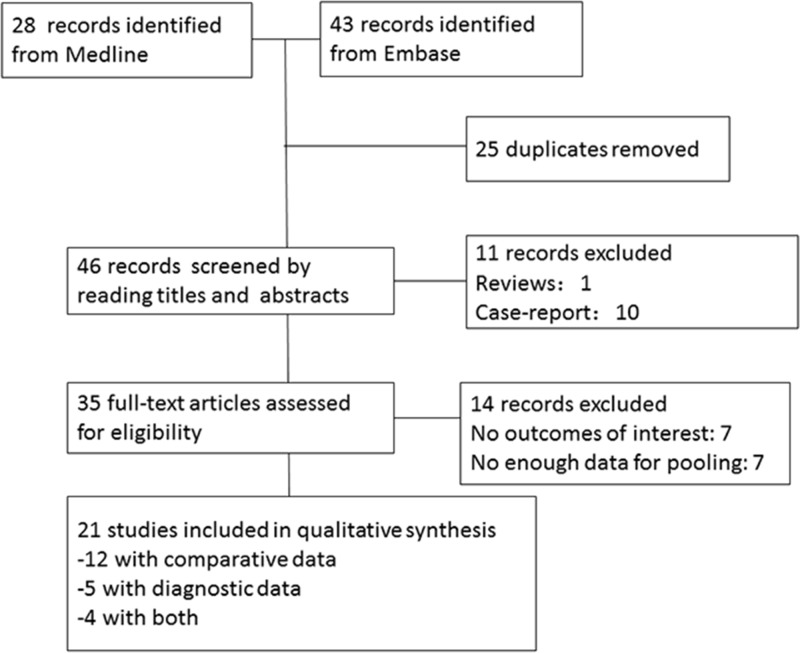
Flow diagram illustrating the systemic literature and study selection process.

The flow diagram of detailed search was illustrated in Fig 1. A total of 71 publications were identified by searching the MEDLINE (28 publications) and EMBASE (43 publications) databases. Of these 71 records, 25 duplications, 1 review and 10 case reports were excluded, leaving 35 records for further assessment. After excluding 14 records due to a lack of either outcome of interests (7 records) or data for pooling (7 records), 21 studies fulfilled selection criteria and were finally included in this systemic review. Details of reasons for exclusion of the studies are also presented in Fig 1.

Characteristics of included studies are presented in Table 1. All of the studies were observational studies, among which 8 studies [12,13,18,19,20,21,22,23] were prospective design. Mean age of study participants ranged from 29.7 to 76.0 years, and the percentage of males ranged from 28.4% to 77.8%. Twelve (57.1%) studies confined the population to patients with AF [13,19,20,21,24,25,26,27,28,29,30,31], with reported percentages of patients with AF ranged from 20%-86% in 7 (33.3%) studies [12,18,22,32,33,34,35], besides, 2 (9.5%) studies focused on patients in sinus rhythm [23,36], 4 studies (19.0%) restricted to the population of patients with mitral stenosis [23,33,35,36], among which, 2 studies reported the prevalence of mitral stenosis ranged from 6.0% to 27.5% [28,37]. Of 21 included studies, 12 (57.1%) studies reported mean or median DD values [13,18,19,24,25,26,27,29,30,32,35,37], 5 (23.8%) studies reported frequency and DD cutoff value on LAT [22,23,31,33,34], and 4 (19.0%) studies reported both [12,20,21,36]. 11 studies have available information to calculate a ratio of the presence of LAT among individuals in the top vs bottom third of DD levels[20,21,22,23,24,28,29,31,33,34,36].

**Table 1:** Characteristics of eligible studies.

Author(ref.)	Year published	Country	Data collection	Sample size	Observational group (n)	Age (years)	Males (%)	AF (%)	MS	Acute stroke	Anticoagulation	Instruments for TEE	Methods for DD measurement	Cutoff of DD (ug/ml)	Excluded population
Black IW, et al.[13]	1993	Australia	Prospective, consecutive	40	SEC/LAT (21)	66	68.9	100	No	No	No	HP21362/3A.Transducer 5 MHz.	EIA(dimertest)	UA	MS, moderate/severe MR or a mitral valve prosthetic.
Yamamura O, et al.[26]	2001	Japan	Retrospective, non-consecutive	19	SEC/LAT (9)	70.8	52.6	100	No	No	No	ATL HDI3000, Philips. Transducer 5 MHz.	LPIA(Datron)	UA	Severe infection, hepatic/renal dysfunction, anticoagulation.
Turchetti V, et al.[32]	2001	Italy	Retrospective, non-consecutive	45	SEC/LAT (20)	72.2	77.8	80	No	No	No	Undefined.	Immunofiltration (NycoCard)	UA	Severe hypertension, lipoidoproteinosis, CRF, DM, severe cerebrovasculopathy, anticoagulation, major valvular disease.
Sato M, et al.[18]	2006	Japan	Prospective, consecutive	43	SEC/LAT (18)	70.8	57.4	22	No	Yes	No	ATL HDI-5000, Philips. Transducer 4–7 MHz.	LPIA(Diayatoron)	UA	PE, infection and DVT.
Topaloglu, S., et al.[35]	2007	Turkey	Retrospective, non-consecutive	46	SEC/LAT (24)	34.0	17.4	39.1	Yes	No	No	Toshiba Ref510 PB, Transducer 5 MHz.	LPIA	UA	Renal/hepatic insufficiency, systemic embolism, LAT, active rheumatism, CHD,DM,DVT, severe MR, malignancy, and hypertension.
Cianfrocca C, et al.[19]	2010	Italy	Prospective, consecutive	159	SEC/LAT (52)	67.0	47.2	100	No	No	No	Vivid 7, GE. Transducer 2.5–3.5–4.0 MHz.	Undefined	UA	Infection, neoplastic, AMI/stroke ≤ 3m, thyrotoxicosis, steroids, NSAIDs, HRT, hematologic, renal/hepatic dysfunction; previous cardioversion.
Jin YY, et al.[25]	2011	China	Retrospective, consecutive	154	SEC/LAT(46)	59.4	59.1	100	No	No	No	Sonos4500/5000, Philips, or Acuson C256, GE. Transducer 2.5–5.0 MHz.	LPIA(Sysmex CA7000)	UA	VHD, AID, infection, active rheumatism, renal/hepatic impairment, malignant, ACS.
Abu-Mahfouz M, et al. [12]	2012	USA	Prospective, consecutive	124	SEC/LAT(21)	55.4	63.7	76	No	No	No	Sonos 5500, Philips. Transducer: undefined.	ELISA(Stago)	0.500	Infection, bleeding, blood transfusion ≤ 1m, surgery, ACS or DVT or PE ≤ 3m, thrombolysis, anticoagulation>7d, malignancy, AID.
Numa S, et al.[24]	2014	Japan	Retrospective, consecutive	468	SEC/LAT(234)	67.0	71.3	100	No	No	No	SSH770A, Toshiba or Vivid E9, GE. Transducer 5 MHz.	Undefined	UA	Acute CVD, infection, taking antihyperuricemic drugs.
Sakai, M., et al.[37]	1994	Japan	Retrospective, non-consecutive	34	LAT(14)	76.0	32.5	100	27.5%	12.5%	No	SA270A, Toshiba. Transducer 5 MHz.	LPIA (Diamondtron)	UA	Anticoagulation, DIC, hepatic disease.
Heppell RM, et al.[29]	1997	UK	Retrospective, consecutive	107	LAT(19)	69.4	64.5	100	No	No	No	Toshiba or Vingmed. Transducer 5–6 MHz.	ELISA (Stago)	UA	RHD, prosthetic heart valve, anticoagulation, haemostatic impairment.
Nakajima K.[27]	2000	Japan	Retrospective, non-consecutive	122	LAT(28)	73.0	62.3	100	No	No	No	HP Sonos500-21363. Transducer: undefined.	Undefined	UA	RHD.
Igarashi Y.[28]	2000	Japan	Retrospective, consecutive	91	LAT(19)	67.0	62.0	100	6%	No	No	SSA270A, Toshiba, Transducer 5.0-MHz.	Undefined	UA	Undefined.
Somloi M, et al.[22]	2003	Hungary	Prospective, consecutive	73	LAT(9)	69.0	52.0	86	No	5%	No	ATL HDI3500, Philips. Transducer: 4–7 MHz.	Roche	0.600	Thromboembolism ≤ 2 m, anticoagulation ≥7 d, equivocal TEE results.
Habara S, et al.[20]	2007	Japan	Prospective, consecutive	925	LAT(83)	68.8	67.4	100	No	No	No	Sonos 2500/5500, Philips. Transducer 2.5–5 MHz.	LPIA (Mitsubishi)	1.150	VHD, prosthetic valve replacement, aortic aneurysm, LVT, AD, DVT and PE.
Sugiura S, et al.[21]	2012	Japan	Prospective, consecutive	225	LAT(23)	62.0	77.8	100	No	No	Yes	Vivid 7, GE. transducer 4–10 MHz.	LPIA (Mitsubishi)	0.125	VHD.
Naresh K, et al.[36]	2014	India	Retrospective, non-consecutive	59	LAT(7)	UA	UA	0	Yes	No	No	Undefined.	Roche	0.400	Undefined.
Rajappa M, et al.[33]	2013	India	Retrospective, non-consecutive	46	LAT(24)	29.7	32.6	83	Yes	No	UA	Undefined.	ELISA kits	4.000	Inadequate TEE results, renal/hepatic dysfunction, infection, neoplastic disease, stroke or PVD.
Kadle N, et al.[34]	2013	USA	Retrospective, non-consecutive	49	LAT(6)	63.0	56.0	52	UA	48%	UA	Undefined.	LPIA	0.200	Undefined.
Yoshida, N., et al.[31]	2015	Japan	Retrospective, consecutive	58	LAT(4)	67.0	56.9	100	No	No	Yes	Vivid 7,GE or iE33,Philips. Transducer: undefined.	LPIA	1.00	Severe MR, MS, and DCM.
Kurakula N,et al.[23]	2016	India	Prospective, consecutive	108	LAT(12)	35.6	28.4	0	Yes	No	No	iE33,Philips. Tranducer: undefined.	Roche	0.510	Moderate/severe MR, AS or AR, AF, renal/hepatic dysfunction, hypertension, DM, CAD, active RHD, infection, malignancy, surgery or trauma, pregnancy, antiplatelet, anticoagulation.

Characteristics of eligible studies.

AF, atrial fibrillation; MS, mitral stenosis; TEE, trans-esophageal echocardiography; DD, d-dimer; SEC, spontaneous echo contrast; LAT, left atrial thrombus; UA, unavailable; MR, mitral regurgitation; CRF, chronic renal failure; DM, diabetes mellitus; PE, pulmonary embolism; DVT, deep venous thrombosis; AMI, acute myocardial infarction; NSAID, non-steroid anti-inflammation drug; HRT, hormone replacement therapy; VHD, valvular heart disease; AID, autoimmune disease; CHD, chronic heart disease; ACS, acute coronary syndrome; CVD, cerebrovascular disease; DIC, disseminated intravascular coagulation; RHD, rheumatic heart disease; LVT, left ventricular thrombosis; AD, aortic dissection; PVD,; AS, aortic stenosis; AR, aortic regurgitation; CAD, coronary artery disease; DCM, dilated cardiomyopathy.

### Risk of bias assessment

**Fig 2:**
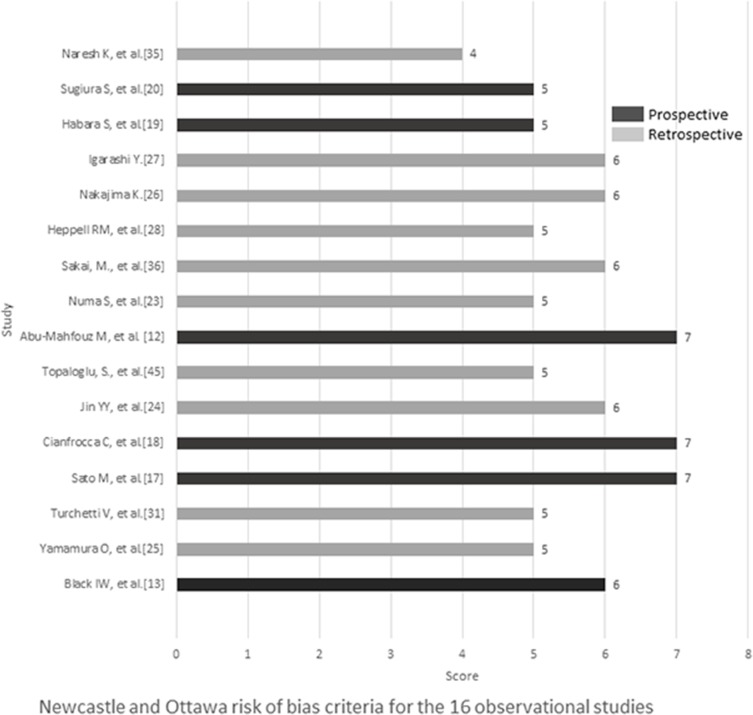
Quality assessment of 16 observational studies by the Newcastle and Ottawa risk of bias criteria.

**Fig 3:**
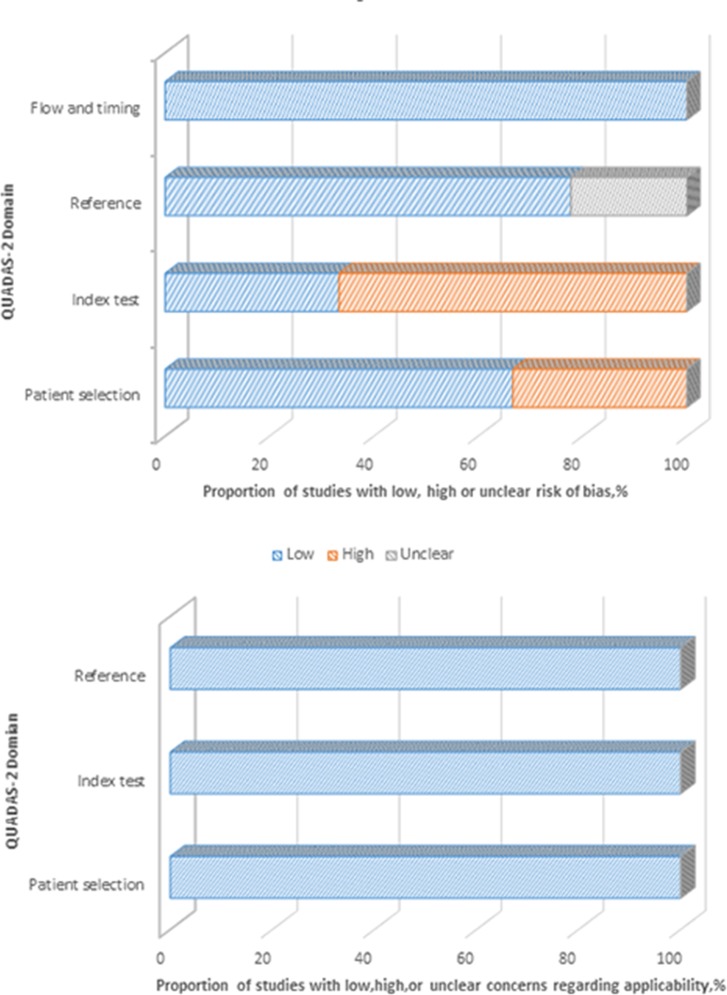
Risk of bias assessment of 9 diagnostic tests by QUADAS-2.

The risks of bias of 16 studies used for pooling SMD of DD levels were assessed via the Newcastle and Ottawa risk of bias criteria [14], in which total scores ranged from 5 to7 with a median of 6 for six prospective studies, and from 4 to 6 with a median of 5 for ten retrospective studies. Four and nine studies had scores equal or higher than median for prospective and retrospective designs, respectively (see Fig 2). Fig 3 presents the risk of bias and concerns regarding applicability of 9 eligible diagnostic test studies via QUADAS-2. In general, three domains for concerns regarding applicability were estimated as “low” risk if the patients included, index test and reference standard used were well matched to the review question. Three studies which were not consecutively or randomly enrolled [33,34,36] and six studies without pre-specified the threshold of index test [20,21,22,34,36] were evaluated as“high”risk of bias in patient selection and index test domain respectively. The reference standard domain of two studies was classified to “unknown” because the authors did not report whether the clinicians were blinded to the index test results when they established the diagnosis [33,34]. Therefore, neither partial nor differential verification bias could be completely excluded.

### Pooled mean d-dimer differences

**Fig 4:**
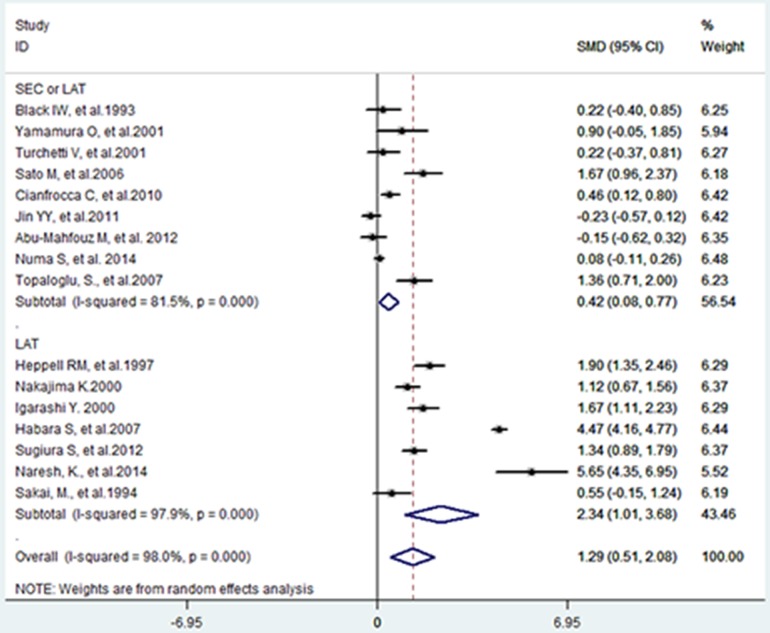
Forest plot of the standard mean difference in d-dimer levels between patients with and without left atrial spontaneous echo contrast/left atrial thrombus.

Nine studies [12,13,18,19,24,25,26,32,35] reported mean DD differences between patients with (n = 445) and without SEC/LAT (n = 644), and seven studies reported the differences between patients with (n = 193) and without LAT (n = 1370), see Fig 4. Data was combined and the estimated total SMD was 1.29 [95% CI: 0.51, 2.08]. This indicated that mean DD in patients with SEC and/ or LAT was 2.29 times higher than mean DD of controls.

Considerable heterogeneity (Chi-square = 765.91, P-value <0.001, I^2^ = 98%) was also observed in the qualitative synthesis. In SEC/LAT subgroup, the estimated SMD was 0.42 [95% CI: 0.08, 0.77], indicating that a slightly elevated DD level existed in patients with SEC/LAT, and the total SMD majorly originated from the LAT subgroup, in which the estimated SMD was 2.34 [95% CI: 1.01, 3.68].

**Fig 5:**
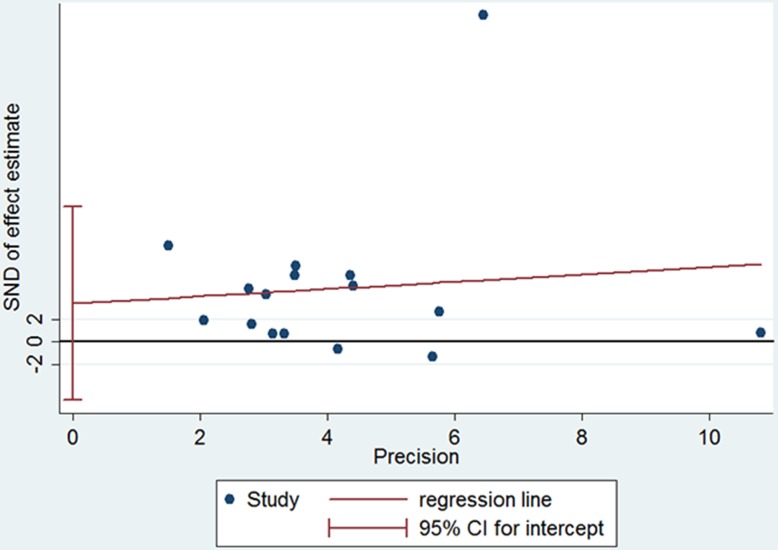
Egger’s test for the assessment of publication bias of 16 observational studies. SND, standard normal deviate.

Besides, a high considerable heterogeneity was also presented in both subgroups (in SEC/LAT subgroup: Chi-square = 43.24, P <0.001, I^2^ = 81.5%; LAT subgroup, Chi-square = 288.35, P <0.001, I^2^ = 97.0%), and thus meta-regression was conducted to explore the possible sources of heterogeneity. Except that sample size was found to be a source of heterogeneity (coefficient = -0.0036, P = 0.033), no significant difference was observed in the coefficient of subgroup (SEC/LAT or LAT), mean age, percentage of males, history of AF, acute ischemic stroke or anticoagulation therapy with the adjusted I^2^ of 84.78%. Funnel plot and Egger’s test revealed no evidence of publication bias (coefficient = 3.3881, P = 0.408). (see Fig 5)

### Association between d-dimer and the presence of LAT

**Fig 6:**
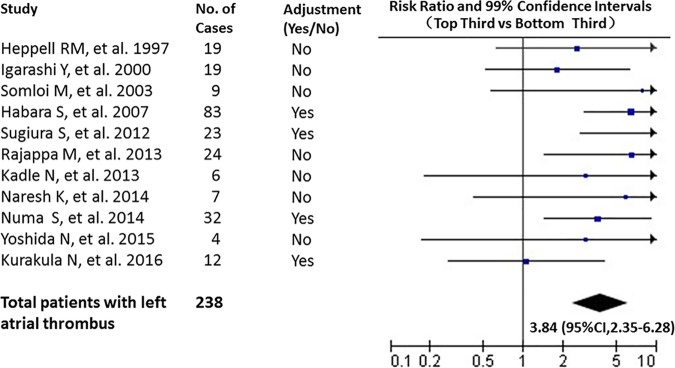
Risk ratios for the presence of LAT comparing the top third with the bottom third based on d-dimer distributions.

**Fig 7:**
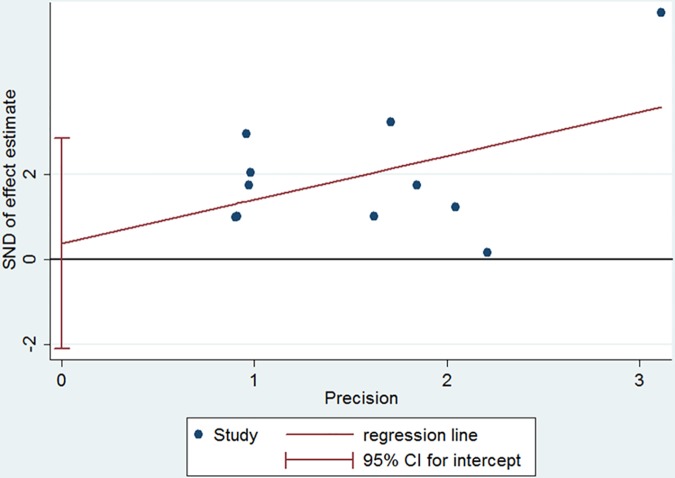
Egger’s test for the assessment of publication bias of 11 studies. SND, standard normal deviate.

Eleven studies [20,21,22,23,24,28,29,31,33,34,36] were included for estimating the association of DD on the presence of LAT. Of 1856 involved patients (weighted mean age of 60.3 years old), 238 patients have TEE identified LAT. Only 4 studies [20,21,23,24] made adjustments for potential confounders. The comparison of individuals with DD levels in the top third vs those in the bottom third yielded a combined risk ratio for the presence of LAT of 3.84 [95% CI: 2.35–6.28] (see Fig 6.), and the estimated mean in these two groups were 1.10 and 0.32 ug/ml. There was no significant heterogeneity among the publish findings of the 11 studies (Q test = 18.45, P = 0.05, I^2^ = 46%). No significant publication bias was exhibited in Egger’s test (coefficient = 0.371, P = 0.741) (see Fig 7.).

### Diagnostic accuracy of d-dimer for the presence of LAT

**Fig 8:**
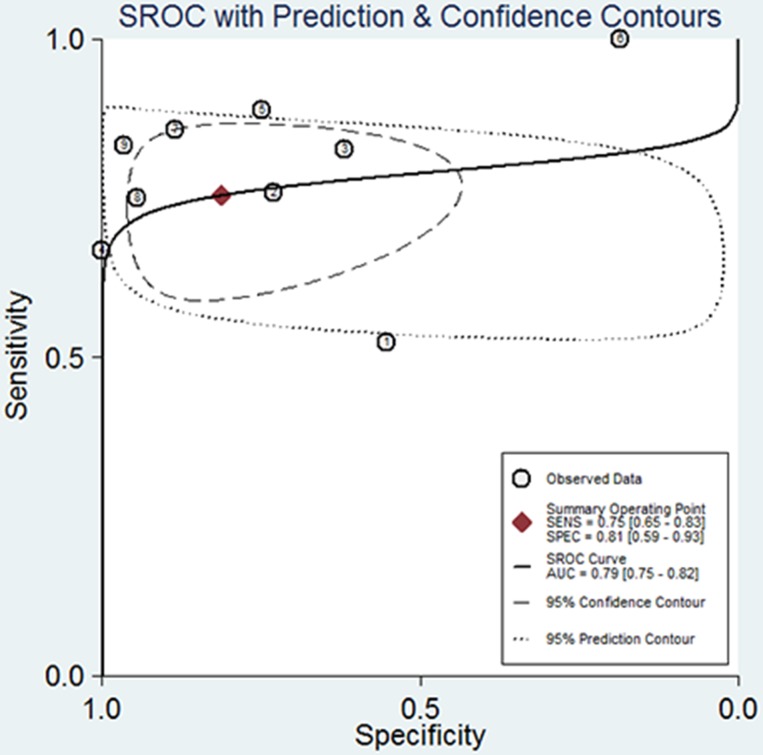
Summary receiver operating characteristic curves (SROC) for overall diagnostic accuracy of d-dimer for the presence of left atrial thrombus. ① Abu-Mahfouz M et al. [12]; ② Habara S et al. [20]; ③ Sugiura S et al. [21]; ④ Rajappa M et al. [33];⑤ Somloi M et al. [22]; ⑥ Kadle N et al. [34]; ⑦ Naresh K et al. [36]; ⑧ Yoshida N et al. [31]; ⑨ Kurakula N et al. [23].

Among the nine studies estimating the diagnostic accuracy of DD for the presence of LAT, five studies were prospective designs. Of 1667 included patients, 189 were diagnosed with LAT. The reported DD cutoff values selected for maximal sensitivity varied across studies, ranging from 0.125 to 4.0 ug/ml. The pooled sensitivity, specificity and positive likelihood ratio were 0.75 [95% CI: 0.65, 0.83], 0.81 [95% CI: 0.59, 0.93] and 4.0 [95% CI: 1.7, 9.9], respectively (see SROC in Fig 8).

**Fig 9:**
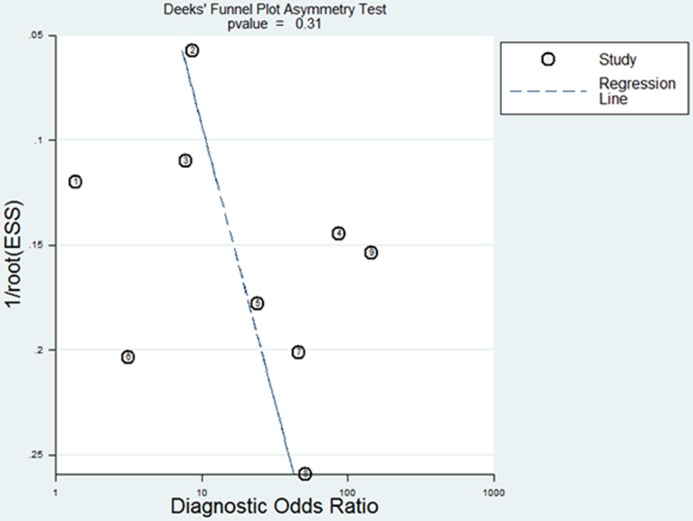
Funnel plot for the assessment of publication bias of 9 diagnostic tests. ① Abu-Mahfouz M et al. [12]; ② Habara S et al. [20]; ③ Sugiura S et al. [21]; ④ Rajappa M et al. [33];⑤ Somloi M et al. [22]; ⑥ Kadle N et al. [34]; ⑦ Naresh K et al. [36]; ⑧ Yoshida N et al. [31]; ⑨ Kurakula N et al. [23].

This pooled result also had a high heterogeneity (Q test = 23.051, P < 0.001, I^2^ = 91%). Meta-regression analysis utilizing a joint model exhibited that DD cutoff values, mean age and percentage of males were the main sources of heterogeneity (Chi-square = 7.42, 14.03, 13.69, respectively; all P ≤ 0.02,). A funnel plot showed symmetry of DD effects without significant publication bias (coefficient = 8.721968, P = 0.309). (see Fig 9)

## Discussion

The present systematic review and meta-analysis investigated the mean DD difference between patients with and without TEE-identified left atrial SEC and/or LAT, and estimated the association between DD levels an the presence of LAT. We found that DD significantly elevated in patients with SEC/LAT, particularly among patients with LAT, and had moderate sensitivity and specificity in identifying the presence of LAT.

Our systemic review majorly included the population of AF and mitral stenosis, which have common pathophysiological characteristics of left atrial dilation and blood stasis, and thus cast high risk of LAT, ischemic stroke and systemic embolism [9,10,19,29]. Left atrial SEC and LAT are TEE-detected risk markers for thromboembolism [8,9,10,13]. Among patients with nonvalvular AF, LAT was observed in 8.8% of patients with sub-therapeutic anticoagulation and 3.6% of patients with sufficient anticoagulation, which was an independent risk factor associated with worse cardiovascular outcomes [38]. Despite adequate oral anticoagulation, patients with AF and dense left atrial SEC still suffer from a substantial likelihood of ischemic stroke and death [39]. Although a high prevalence of left atrial SEC has been found in patients with LAT [10], we identified that DD levels in patients with LAT were substantially higher than those with SEC. Several mechanisms were involved in the pathological conditions of left atrial SEC, including blood stasis and erythrocyte aggregation, which increases blood viscosity, leading to a propensity of thrombosis [13]. However, previous study had demonstrated that SEC was not an indicator of platelet activation or fibrin formation [13]. In addition, the determination of left atrial SEC in different studies might be hard to harmonize, which depended more on the experience of diagnosticians than LAT. Among consecutive patients with AF, although Numa S., et al.[24] and Habara S., et al.[20] reported a similar prevalence of LAT (8.5% and 8.9%, respectively), the prevalence of left atrial SEC was significantly varied (49.8% and 14.3%, respectively). These reasons could potentially explain the significant heterogeneity of DD levels among the individuals with left atrial SEC in the subgroup analysis.

The elevated DD level, likewise, was related to the risk of stroke and death, and adds to the predictive value of clinical adverse events in anticoagulated patients with AF [40]. Previous meta-analysis indicated that a positive DD test was associated with an increasing risk of stroke and systemic embolism [7,41], reversely, a negative DD test result was valuable for excluding thrombosis and thromboembolism in patients with DVT and PE [42,43]. Moreover, high graded left atrial SEC was related to increased DD levels in patients with nonvalvular AF [20]. Similar to these studies, our meta-analysis also exhibited considerable association between elevated DD levels and SEC/LAT, particularly among patients with LAT. The ratio risk of the presence of LAT among individuals with DD levels in the top third was about 4 times higher than those in the bottom third.

DD may be a potential valuable marker for diagnosing LAT. In our study, the pooled specificity of DD test was 0.81 [95% CI: 0.59, 0.93], indicating that a normal DD level has moderate to high potential to rule out LAT. Therefore, a negative DD value may be helpful to identify low-risk patients for the presence of LAT or thromboembolism, and to reduce unnecessary image screening for them. In addition, a positive DD value also exhibited moderate sensitivity in the prediction of LAT (0.75 [95% CI: 0.65, 0.83]), which suggested a potential diagnostic applicability of DD. Therefore, a positive DD value may be a complement to CHA2DS2-VASc score [44], and contribute to identify high-risk patients with SEC/LAT for anticoagulation therapy. It is noted that all of the included studies have ruled out other conditions associated with fibrin formation and degradation, such as infection, inflammation, malignancy and thrombus other than left atrium, etc.(see Table 1) Thus, one should be cautious to extensively apply the results to other population or situations.

There are several strengths in our present study. To our knowledge, this is the first systemic review and meta-analysis to evaluate the relationship between DD levels and the presence of left atrial SEC/LAT. A comprehensive search of literatures from 2 main databases were approached, and two reviewers independently selected the relevant studies and extracted the data, and also risk of bias was estimated by other 2 independent reviewers. The reference standard was determined by TEE detection, which is a gold standard test for the diagnosis of left atrial SEC/LAT. The pooled sample size was large enough to estimate the mean DD difference between two groups, and verify the association and the diagnostic accuracy of DD on the presence of LAT. Subgroup analyses and meta-regression were also performed to explore the source of heterogeneity. In addition, no significant publication bias was found.

There are a few limitations inherent to this meta-analysis. First, heterogeneity was high in all pooled analyses even after associated factors were adjusted. Our meta-regression analysis indicated that sample size was a source of heterogeneity (coefficient = -0.004, p = 0.033) when pooling SMDs of16 included studies. Some of the included studies were small in size (≤100 cases, 8/16) and 6 studies enrolled patients non-consecutively (6/16). Second, more than 5 kinds of DD assays and various cutoff values (range from 0.125 to 4.0 ug/ml) were used across the included studies. Although different DD assays displayed very similar tendency [45], our meta-regression indicated that cutoff values and mean age contributed to a part of the sources of heterogeneity. Moreover, a large prospective diagnostic management outcome study has demonstrated that age-adjusted DD cutoff was superior to a fixed one to rule out thromboembolism [46]. However, without individual patient data from each study, it was difficult to distinguish and calibrate the optimal cutoff values of DD. Third, due to the study design of the original included studies, the causal relationship could not be concluded between DD and SEC/LAT. Another potential bias is the absence of clear policy regarding antithrombotic treatment regimens and the lack of evaluation of such treatment on DD cutoff values. In addition, some key clinical endpoints, such as ischemic stroke and systemic embolism, which were thought to be the major risk of SEC/LAT, were not included in our meta-analysis. Future studies should encompass well-designed prospective cohort studies with balanced age, standardized DD assays and an uniform cutoff value, and be required to clarify the following two questions: ① if DD is temporally associated with the development of LAT, and ②if DD-guided anticoagulant therapy has a favorable impact on thrombosis and thromboembolisms.

## Conclusions

Notwithstanding the limitations of observational studies and indirect data, this systematic review demonstrated that DD elevated among patients with SEC/LAT, particularly in those with LAT. DD levels had moderate sensitivity and specificity for diagnosing LAT in conditions leading to left atrial stasis, such as AF and mitral stenosis. These findings suggest that DD may benefit the risk stratification of patients with atrial fibrillation, providing further evidence for anticoagulation therapy among patients susceptible to left atrial SEC/LAT.

## Supporting information

S1 File PRISMA checklist for the present study. (DOC)
